# A new flow chip in combination with multiphoton microscopy as a protocol for longitudinal 3D imaging of tissue calcification under shear stress

**DOI:** 10.1002/2211-5463.70295

**Published:** 2026-06-22

**Authors:** Vytautas Kučikas, Aaron D. Morgan, Ignas Čiurlionis, Christian Böhm, Stefan Jockenhövel, Willi Jahnen‐Dechent, Marc A. M. J. van Zandvoort

**Affiliations:** ^1^ Department of Genetics and Cell Biology, Research Institute for Oncology and Reproduction (GROW) Maastricht University the Netherlands; ^2^ Institute for Molecular Cardiovascular Research (IMCAR) RWTH Aachen University Hospital Germany; ^3^ Helmholtz‐Institute for Biomedical Engineering, Biointerface Lab RWTH Aachen University Hospital Germany; ^4^ Science College, Faculty of Science and Engineering Maastricht University the Netherlands; ^5^ Department of Biohybrid & Medical Textiles (BioTex), AME‐Helmholtz Institute for Biomedical Engineering & Institut für Textiltechnik (ITA) RWTH Aachen University Germany

**Keywords:** calcification, flow chip, live imaging, multiphoton

## Abstract

We present a novel miniaturized flow chip platform enabling thin‐layer smooth muscle cell (SMC) culture, continuous tangential perfusion, dual‐channel nutrient control, and longitudinal subcellular resolution 3‐D imaging via multiphoton microscopy. Using a case study of longitudinal imaging of vascular calcification, a key pathological process in cardiovascular disease typically challenging to dynamically model under physiological flow, we describe the platform and its potential. Within this system, immortalized vascular SMCs embedded in fibrin hydrogel on a textile‐reinforced scaffold are exposed to unidirectional flow (12 mL·min^−1^) of calcification medium for 4 days, with and without supplemental Mg. Fluorescent tracking of mineral deposition (mRuby‐labeled fetuin‐A) and cytoplasmic dyes (CellTracker Green) enable daily volumetric imaging. In Mg‐free conditions, rapid mineralization initiates at the textile scaffold and engulfs the construct by day 2, plateauing thereafter, yet viable cells remain postcalcification. In contrast, Mg‐supplemented constructs show markedly suppressed calcium phosphate deposition, progressing only slowly over the time course. These longitudinal findings align with known Mg‐mediated inhibition of hydroxyapatite formation and cellular osteogenic transition. Thus, this case study highlights a platform which can reproduce shear‐flow conditions, modulating media ionic composition, and performing time‐lapse 3‐D imaging of live cells within a flow environment. This versatile system not only has uses for further mechanistic studies of vascular calcification, screening of anticalcification therapies, and adaptation to endothelialized co‐cultures or excised natural tissues, but also has potential for longitudinal model studies in a broader context.

## Graphical abstract

Miniaturized flow chip platform enabling continuous perfusion and longitudinal multiphoton 3D imaging of vascular smooth muscle cell constructs under physiological flow. Brightfield imaging guides region selection, while CellTracker Green and mRuby‐labeled fetuin‐A visualize cells and mineral deposition, respectively. Magnesium supplementation markedly suppresses calcification compared with Mg‐free conditions over 4 days of culture.

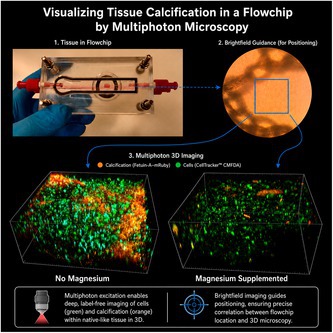

**Abbreviations**
PC: polycarbonate
PMMA: poly‐methyl‐methacrylate (acrylic)
SMC: smooth muscle cell
SNR: signal‐to‐noise ratio

Vascular calcification is a complex multivariable effect and a crucial factor in vascular disease [1]. However, the calcification process is challenging to predict and model [2]. To effectively study the process of calcification in human vascular model systems, there is a need for the combination of three different aspects: (1) physiological pressure and flow conditions [2, 3, 4], (2) mimicking of the vascular nutrition perfusion, and (3) subcellular resolution imaging longitudinally in time. We present a platform that is capable of all three of these aspects. It consists of a flow chip for a thin (boundary) cell culture, in which flow conditions like those under physiological conditions can be applied. Furthermore, it contains two channel capabilities for specific nutrient conditions on both sides of the samples and longitudinal subcellular tracking using multiphoton microscopy. In this experiment, we show the potential of this system to track calcification in 3D cell culture of smooth muscle cells (SMCs) [5, 6]. We will describe the protocols needed for sample preparation and imaging experiments and conclude that the system can be used effectively to study the calcification process longitudinally. We discuss the possibility of expanding investigation of calcification to endothelialized SMC cultures or even native tissues from human or animal origin. Furthermore, we discuss how adapting the protocols and procedures described here for this specific application, our system can be applied to high‐resolution imaging in a broader set of different kinds of samples where shear stress [7, 8, 9, 10] and tracking in time are required.

## Materials

### Cell culture, medium preparation, and biohybrid construct preparation

Immortalized vascular smooth muscle cells (clone IM3) were kindly provided by Claudia Goettsch (RWTH Aachen University Hospital). The generation of immortalized cells is fully explained in a previous study [11]. Cells were cultured in Dulbecco's modified Eagle medium (DMEM, 31053‐044, Lot: 203043; Gibco) supplemented with 10% fetal bovine serum (FBS, P30‐3033, Lot: P231101, Pan Biotech) and 1% Penicillin–Streptomycin (PenStrep, 15 140‐122, Lot: 203043; Gibco). The calcification medium (CM) contained a total calcium concentration of 4.1 mm and total phosphate concentration of 3.0 mm. For experiments with magnesium supplementation, the total magnesium concentration was adjusted to 5 mm.

The textile‐reinforced biohybrid scaffold was fabricated from a tubular warp‐knitted polyethylene terephthalate (PET) mesh (Tuell‐filet, 24 filaments, E15; ICF Mercantile LLC, NJ, USA) with a diameter of 22 mm [12]. The mesh was manually cut into 20‐mm circular discs to fit the flow chip chamber. Each textile disc was embedded in a fibrin‐based hydrogel containing a VP‐co‐GMA copolymer to provide mechanical support and biocompatibility. IM3 cells were seeded onto the scaffold at a density of 5 × 10^5^ cells·mL^−1^ in 5 mL of medium. After reaching sufficient cell density, the constructs were mounted in a flow chamber and subjected to continuous unidirectional flow (12 mL·min^−1^) of calcification medium. The constructs were maintained under flow for 4 days, except during periodic imaging sessions, and were finally extracted from the flow chip for high‐resolution microscopic analysis.

### Miniaturized flow chip system

The development of the geometry of the flow chip is described in detail in another publication [13]. In short, the flow chip was designed to provide a uniform laminar flow profile across the observed region. Two polymers were used for cost‐effective and simple manufacturing using CNC milling machine: polycarbonate (PC) and acrylic (PMMA). The base pieces, holding the sample in place and providing the media from the bottom side, were milled from PC, which has superior structural properties compared to PMMA and provides sufficient structural integrity. Also, sterilizing PC parts is much easier, as the melting temperature and chemical resistance allow both autoclaving and ethanol sterilizations. On the downside, PC has a refractive index of 1.58, which exceeds the range of refractive indexes of the materials commonly used in microscopy workflows. Therefore, there are no standard objectives commercially available that could properly focus in such optically dense media. For this reason, the top part of the chamber was manufactured using PMMA, which, despite having poorer structural and chemical resistances, provides superior optical clarity and a refractive index of 1.48, which is in the range of objective adjustability.

### Controlling volumetric flow

A peristaltic pump was used to provide 12 mL·min^−1^ volumetric flow rate of medium drawn from a closed‐loop reservoir. The geometry of the flow channel was designed such that this amount of flow will apply approximately 10 dyne/cm^2^ to the mounted sample surface, which is in the range of physiological stress levels in arteries [14, 15, 16]. While the whole sample is 22 mm in diameter, the flow‐induced shear stress was limited to the 4 × 5 mm area in the middle. The flow profile and induced shear stress were evaluated using iterative methods using Computational Fluid Dynamics simulations of autodesk cfd 2019 software. Silicone tubing and polymer connectors were used to connect the chip to the pump and reservoir. The filtered gas perfusion was possible via filtered gas exchange at the liquid interface inside the reservoir. About 30 mL of cell medium was used in the system, while the reservoir total volume was 50 mL.

## Methods

### Fluorescent labeling

Calcification visualization was enabled using the plasma protein fetuin‐A, which binds calcium phosphate mineral, genetically fused to the fluorescent protein, mRuby3 [17, 18, 19]. Calcification staining was achieved by adding 10 μm fetuin‐A‐mRuby3 to the calcification medium. Cellular content was labeled using CellTracker™ Green CMFDA (C7025; Thermo Fisher Scientific GmbH, Dreieich, Germany), which was used once on the cells just before seeding them in the fibrin gel. The concentration of 10 μm of CellTracker was used. On the last day of the experiment, relabeling with 10 μm CellTracker in addition to nuclei staining Hoechst 33342 (5 mm, #H1399; Invitrogen GmbH, Darmstadt, Germany) was used to control the fading of the labels.

### Imaging sessions

Every day of the in total 4‐day experiment, the flow chip system was decoupled from the pump, brought to the multiphoton microscope, used for imaging, brought back and reconnected to the pump. Imaging sessions were kept short within 15 min, to keep the total decoupling time under 30 min.

### Multiphoton microscopy

Multiphoton microscopy was carried out using the Leica STELLARIS 8 DIVE FALCON multiphoton microscope (Leica Microsystems GmbH, Wetzlar, Germany), equipped with Spectra Physics Insight X3 femtosecond laser. Images were acquired using OLYMPUS XLPlan N 10×/0.60 SV MP objective with a wide‐range RI correction collar. This objective was specifically selected due to an exceptional working distance of 8 mm. The objective used with water immersion and correction collar was set empirically to maximize signal‐to‐noise (SNR) ratio. An excitation wavelength of 1000 nm was used with 10 mW excitation power at the focal plane, while the detection ranges were set at 490–505 nm for CellTracker green and 510–570 nm for Fetuin‐A‐mRuby3. On the last day of the experiment, after relabeling of the samples, the excitation wavelength was set to 800 nm with 20 mW power at the sample plane and detection ranges were set to 405–425 nm (Hoechst), 433–488 nm (CellTracker), and 510–570 nm (Fetuin‐A‐mRuby3). For every timepoint, a volume of 1107 × 1107 × 400 μm was imaged with voxel size of 0.54 × 0.54 × 4 μm. During the imaging under the microscope, the samples were kept in 37° C, by the temperature control unit and custom enclosed microscope stage (Okolab Inc. Pozzuoli, Italy).

### Image analysis

Image analysis was carried out using imagej (1.54p) software. Acquired z‐stack images were manually registered using persistent internal tissue structures as anchoring points. The z‐stacks were uniformed by trimming the exceeding volumes and then maximum intensity projections were derived. We found maximum intensity projection images easier to compare for our samples; however, in cases of denser tissues with clear morphological features 3D renderings might be the preferred choice.

## Tips and Tricks/troubleshooting

### Flow chip preparation for microscopy

**Fig. 1:**
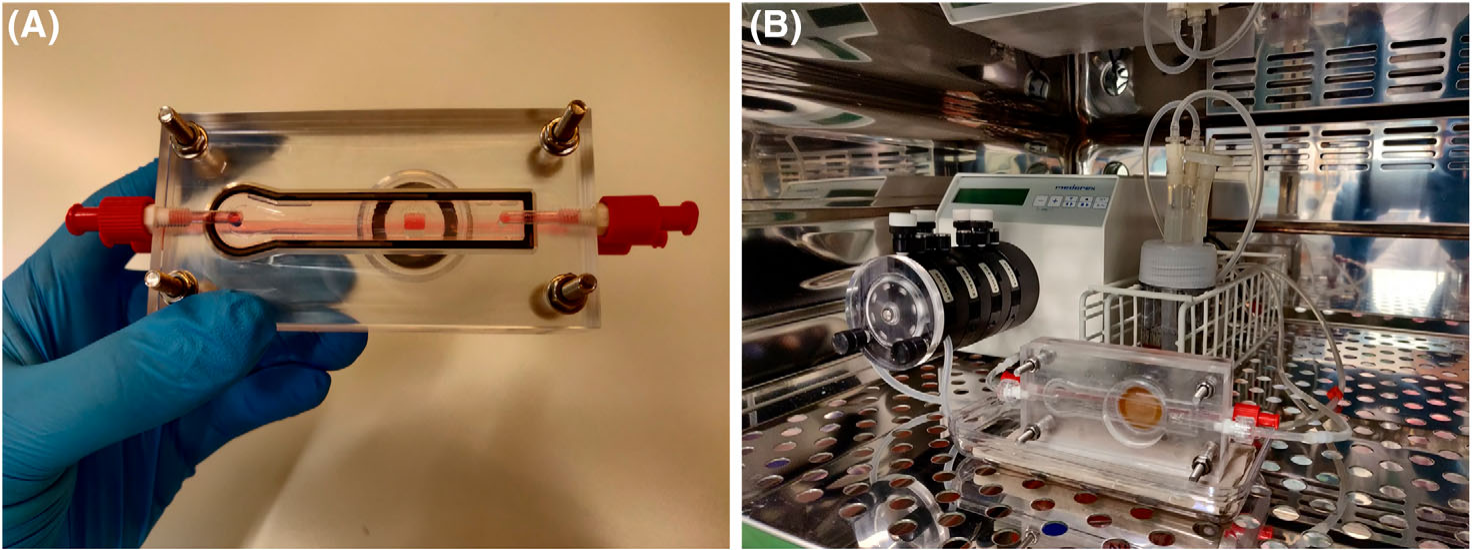
Flow chip designed for cell experiments and live imaging. (A) Assembled flow chip, held in hand for scale. (B) Flow chip in a complete experiment setup with 2 fluid reservoirs and a peristaltic pump.

The most challenging part of setting up multiphoton microscopy on the flow chip was configuring the chip hardware to enable effective multiphoton excitation. First, a delicate balance had to be achieved between maintaining the structural integrity of the flow chip, while keeping the top layer sufficiently thin to accommodate the limited 8 mm working distance of the microscope objective. After several trials, we arrived at a plate design with a cutout large enough to insert the objective close to the sample, while the surrounding edges preserved the necessary mechanical stability (Fig. 1). The remaining PMMA thickness between the objective and the sample was 5 mm. Furthermore, although the optical surfaces were initially machined using standard CNC equipment, the resulting surface finish was too rough and insufficient for efficient multiphoton excitation. Therefore, these surfaces were subsequently polished to reduce scattering, which led to a noticeable increase in signal intensity.

### Labeling

We went through a rigorous process to optimize the labeling protocol on monolayer cultures of the SMCs. On one hand, sufficient label concentration was required to achieve a strong signal. On the other hand, introducing labels into living samples risks disturbing cellular physiology [20, 21]. For example, we tested *in vivo*–compatible nuclear dyes such as Hoechst 33342. Although this dye is not inherently toxic, prolonged exposure to excitation light caused significant stress to labeled nuclei, leading to a high rate of apoptosis and hence limited division of cells [22]. Hoechst 33342 hence does not allow undisturbed longitudinal imaging. Calcein, MitoTracker, and Syto family labeling were also tested with different concentrations, but all led to a significant number of apoptotic cells and were discarded for being too harmful for long term experiments. We therefore adopted a more specific labeling approach, using only dyes that do not bind to vital cellular organelles.

Two fluorescent dyes were used in our experiments. To visualize cellular content, we employed CellTracker™ Green CMFDA, which becomes fluorescent after intracellular metabolism and remains trapped as a byproduct in the cytoplasm. For our focus, that is, imaging of the calcification, we used Fetuin‐A‐mRuby, which binds calcium phosphate minerals deposited in the extracellular space, thus avoiding disruption of cellular homeostasis. While Fetuin‐A‐mRuby was continuously supplied through the circulating flow medium during the experiment, CellTracker was applied only at discrete time points. Initially, cells were stained with CellTracker before embedding them in the fibrin gel, improving dye distribution and avoiding diffusion‐related probe penetration issues. The drawback of this approach is that with each cell division, the CellTracker signal intensity in the daughter cells effectively halves, making cells progressively harder to distinguish over time. However, for our purposes of imaging over the course of 4 days, this method performed reasonably well: cells remained detectable after 4 days of incubation, aided by an increase in excitation intensity during the later stages of the experiment. Finally, on the last day, we performed additional staining with CellTracker, complemented by the addition of Hoechst 33342. This combination at the end stage of the experiment enhanced the signal in actively proliferating cells and provided clear visualization of cell nuclei. In the same way, the autofluorescence approach was tested. While it did not produce a detectable signal in the safe excitation power range, it also worked as a negative control for our final labeling choice.

### Imaging

**Fig. 2:**
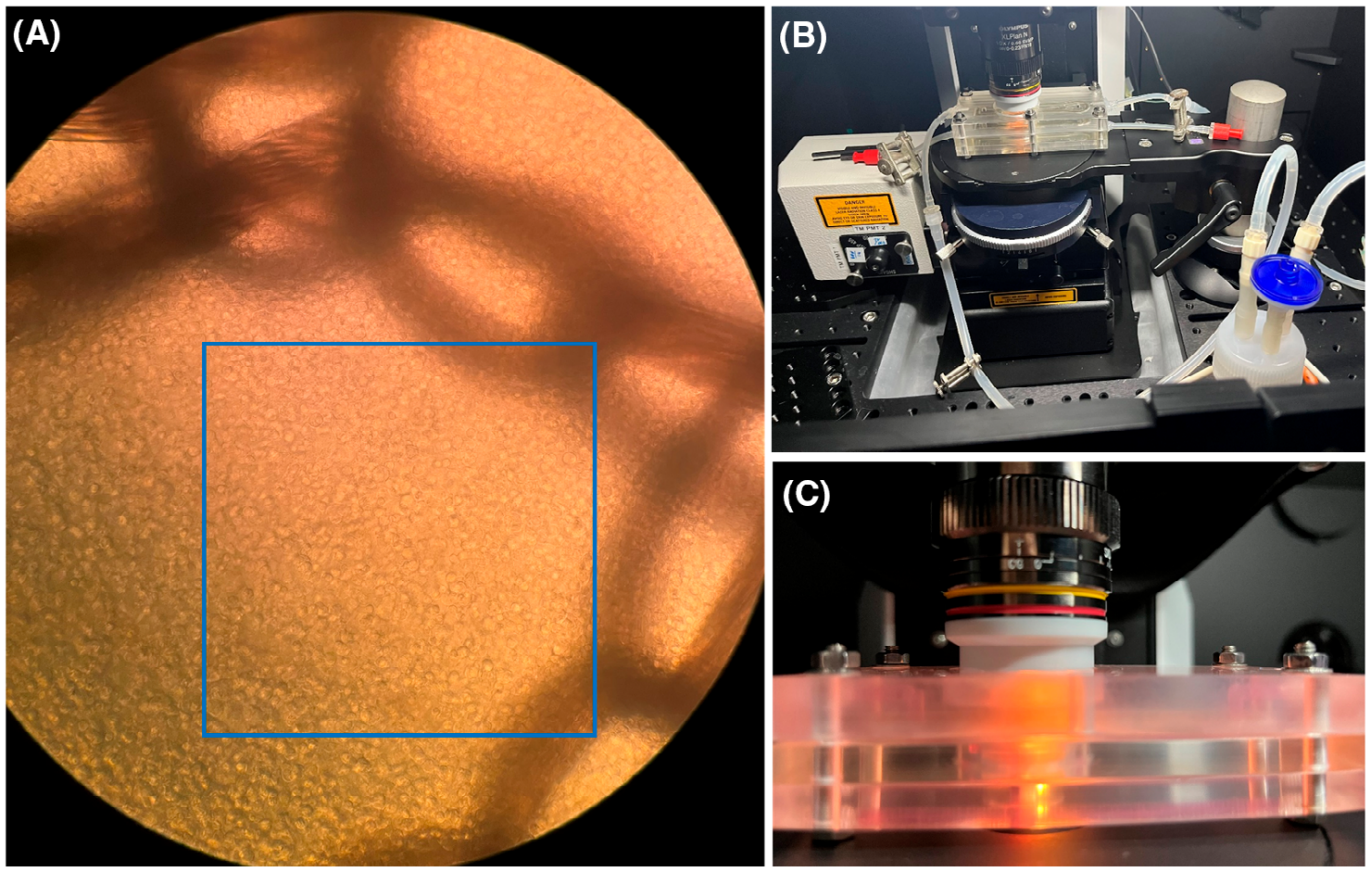
(A) Brightfield ocular view used to guide the correct FOV selection. Selected FOV for multiphoton imaging is marked with the square. The textile scaffold of the sample is used as a marker to consistently follow the same sample region. (B) Multiphoton microscope imaging setup. (C) Detailed image of the flow chip under the microscope objective in the imaging position.

**Fig. 3:**
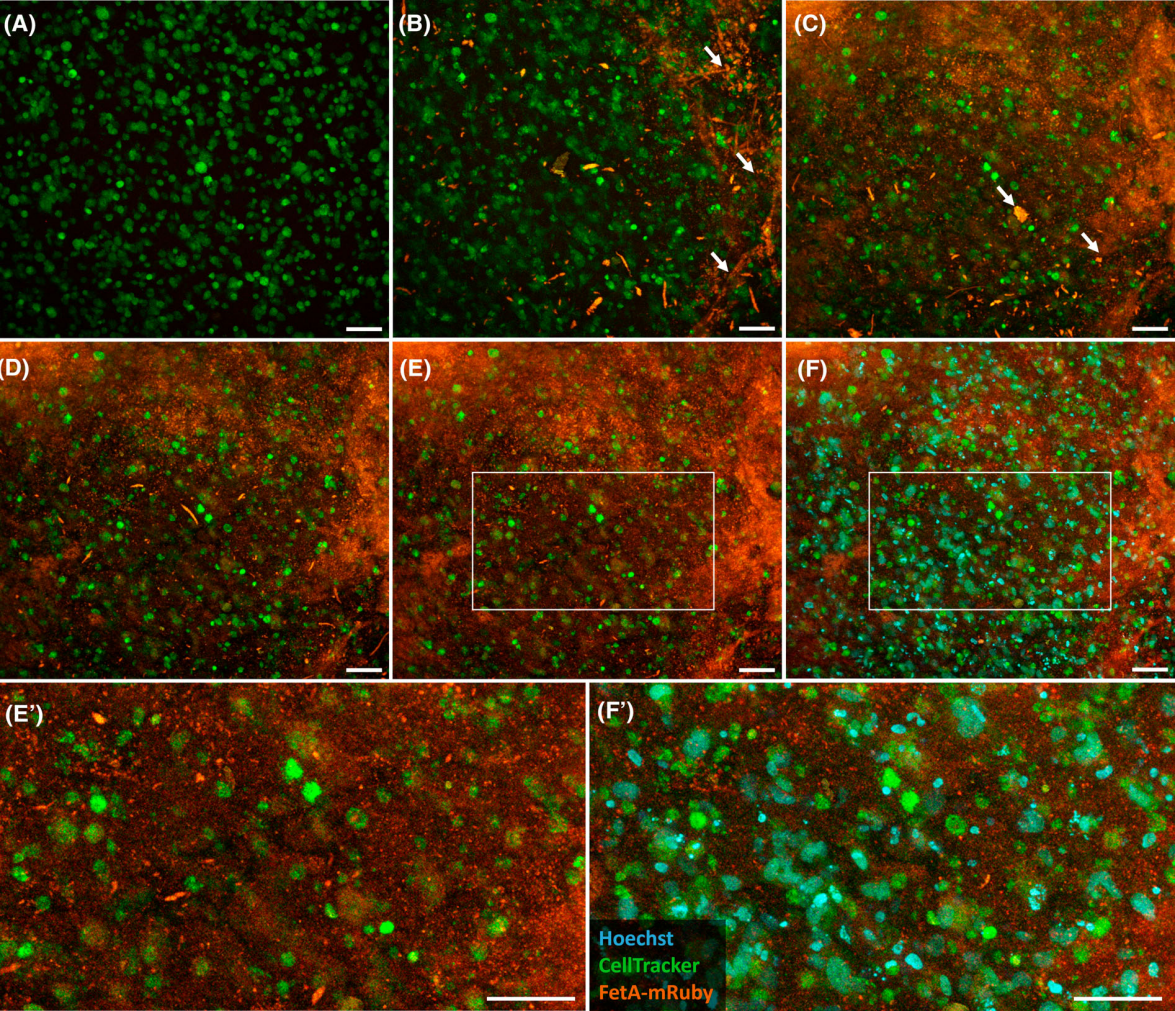
Maximum intensity projections (through the whole thickness) of the selected FOV of the samples in the flow chip with calcifying media. (A) Day 0 timepoint, imaged directly after assembling the sample into the flow chip. (B) Day 1 of the experiment. Visible calcification on the textile scaffold (B, arrows). (C) Day 2 of the experiment. Calcification spread from the textile through the whole sample. Some larger deposits precipitated on the surface of the sample (C, arrows). (D) Day 3 of the experiment. Abundant calcification throughout the whole sample. Some of the previously visible surface deposits were flushed away by the medium flow. (E) Day 4 of the experiment. No visible changes compared to previous day, suggesting that calcification process reached a plateau due to calcium depletion in nutrition media. (F) Day 4 of the experiment, after relabeling with CellTracker and Hoechst. Compared to E, it revealed actively proliferating cells and nuclear content. (E′, F′) Zoomed in images of the indicated areas of E and F. Labeling: CellTracker Green CMFDA (green), Fetuin‐A‐mRuby (red) and Hoechst 33342 (blue, F and F′ only). Scale bars = 100 μm.

**Fig. 4:**
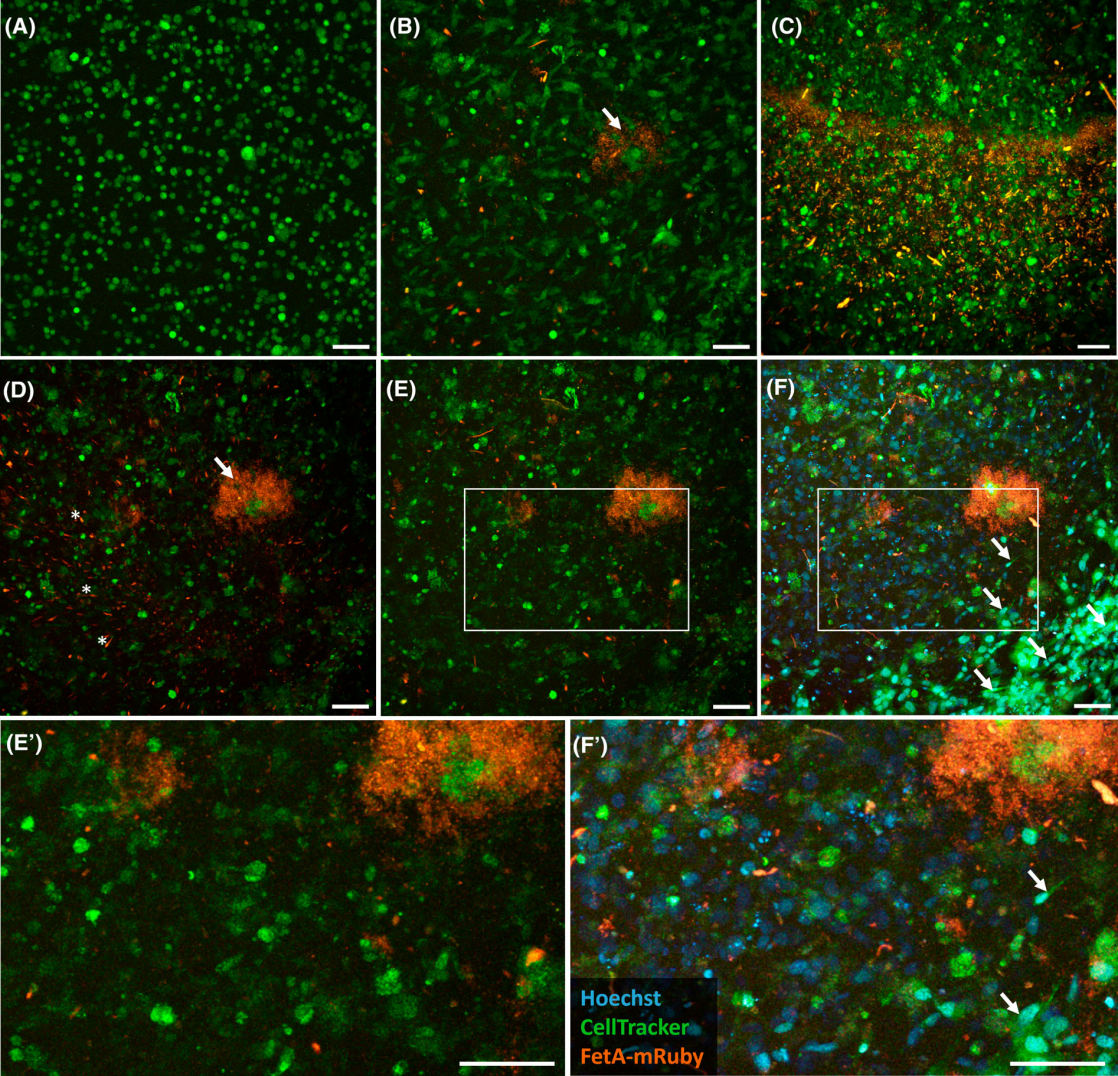
Maximum intensity projections (through the whole thickness) of the selected FOV of the samples in the flow chip with calcifying media and additional magnesium. (A) Day 0 timepoint, imaged directly after assembling the sample into the flow chip. (B) Day 1 of the experiment. Visible calcification deposit on the surface of the sample (B, arrow). (C) Day 2 of the experiment. More surface calcification deposits. Imaging location selected incorrectly (see Tips and Tricks/Troubleshooting). (D) Day 3 of the experiment. Same large surface deposit visible (D, arrow), additional surface deposits appeared (D, asterisks). (E) Day 4 of the experiment. No significant changes compared to previous day, suggesting strong calcification inhibition caused by magnesium supplementation. (F) Day 4 of the experiment, after relabeling with CellTracker and Hoechst. Compared to E, it revealed actively proliferating cells and nuclear content. Especially active cells on the right‐side bottom corner (F and F′ arrows), where the cells had proximity to the textile scaffold, suggesting that increased mechanical stability allowed the cells to proliferate more actively. E′ and F′—zoomed in images of the indicated areas of E and F. Labeling: CellTracker Green CMFDA (green), Fetuin‐A‐mRuby (red) and Hoechst 33342 (blue, F and F′ only). Scale bars = 100 μm.

The imaging workflow consisted of two steps: (1) selecting the field of view and (2) acquiring the image stack. For the first step, brightfield imaging through the microscope ocular was used (see Fig. 2A). Since the samples contained a textile scaffold for mechanical reinforcement, its pattern served as a reference for locating the same position during repeated acquisitions. This approach provided reasonably good positioning in the xy‐plane and allowed longitudinal imaging of the same area (Fig. 3). However, due to the periodic nature of the textile pattern, selecting the exact same location was not always straightforward. For example, in Fig. 4C, the xy‐position was chosen incorrectly, missing the large surface calcification structure visible in Fig. 4B,D. Unfortunately, such discrepancies often become obvious only in retrospect—during acquisition, it is difficult to determine whether the field of view is incorrect or whether the sample morphology has changed.

In addition, although xy‐translation was generally well reproduced, rotation around the *z*‐axis was not stabilized. Consequently, although the acquired images were very similar, they could not be directly overlaid for precise comparison. To improve reproducibility and allow such direct overlay, it would be advisable to incorporate fixed reference points—either mechanically (e.g., defined support positions for the flow chip) or within the sample itself (e.g., integrated fluorescent landmarks or beads).

For z‐positioning, multiphoton microscopy was used to identify the upper and lower surfaces of the sample and select the midpoint as the reference focal plane. Over the course of the experiment, the sample thickness slightly decreased due to contraction, making a stable *z*‐axis reference essential. Fortunately, in our case, the penetration depth of multiphoton microscopy allowed imaging through the entire sample, simplifying this step.

For step (2), the image stack acquisition was performed with a focus on preserving sample viability. Bidirectional scanning was employed to reduce overall imaging time. To improve the SNR, four frame repetitions were averaged instead of increasing excitation power or reducing scanning speed, both of which would lead to higher local thermal stress. In general, repeated scans with frame averaging/accumulation are preferable to prolonged single‐pixel exposures or increased excitation power, provided that the minimal SNR required for data collection and analysis is achieved.

We used 10 mW excitation at 1000 nm, which in these optical conditions was sufficient to provide detectable signal, while also proved to be not phototoxic to the cells. Although multiphoton microscopy is inherently less damaging to live samples than other fluorescence imaging modalities (e.g., widefield or confocal microscopy), this approach further minimized phototoxicity. On the final fourth day, when the experiment was nearing completion and phototoxicity was no longer a major concern, the samples were imaged using higher excitation power at a different wavelength (800 nm instead of 1000 nm). This adjustment was necessary due to the additional Hoechst labeling at that day, a probe that cannot be excited at 1000 nm. However, this change of wavelength reduces the excitation efficiency for the other fluorophores, necessitating the increase in laser power. In general, for different sample types or labeling protocols, imaging parameters should be re‐optimized to balance signal quality and sample preservation.

The total imaging routine was designed to be completed within 30 min, since medium flow was paused during imaging and gas exchange was limited. Previous tests showed that the system remained stable for up to 30 min outside the incubator without inducing cellular stress, if temperature was maintained at 37 °C. The image acquisition workflow was designed with this limitation in mind: The pixel size was set to match theoretical resolution, effectively resulting in slight undersampling relative to the Nyquist criterion. Under typical conditions, sample setup, field‐of‐view selection, and image stack acquisition, the total experiment time required up to 15 min, allowing the entire procedure—including sample handling and imaging—to remain well within the predefined 30‐min limit, with additional time available if needed.

## Discussion

We present a case study using the flow chip platform to longitudinally follow the calcification process of tissue‐engineered patches. These patches, composed of smooth muscle cells embedded in fibrin gel and reinforced with electrospun textile, were exposed to shear stress generated by the continuous tangential flow of nutrient medium. Calcification of the patches was imaged daily for 4 days under two conditions: with and without magnesium supplementation.

In the absence of additional magnesium, calcification progressed rapidly. The textile scaffold appeared to serve as a nucleation site for mineralization (Fig. 3B), from which calcium phosphate deposits propagated throughout the sample. By the second day, the entire construct was calcified, with no major changes observed in subsequent days (Fig. 3C). This plateau may have resulted from depletion of calcium and phosphate in the calcification medium, as these ions were likely exhausted during mineral deposition. After four full days of the experiment, the sample was relabeled with Hoechst and CellTracker dyes (see Methods), revealing that viable cells remained despite the extensive calcification (Fig. 3F,F′).

In contrast, samples treated with magnesium‐enriched medium exhibited markedly suppressed calcium deposition. Although some mineral accumulation was still detectable, it was substantially less abundant than in magnesium‐free medium conditions (Fig. 4 in comparison with Fig. 3). In this case, the observed field of view included a large surface deposit that began forming on the first day (Fig. 4B, arrow), along with several smaller deposits that appeared at later stages but were not in correlation with textile positioning. In contrast, increased cell proliferation was observed around textile reinforcement, suggesting that textile‐provided stability promotes faster cell proliferation (Fig. 4F). These findings suggest that while prolonged culture in magnesium‐containing medium might eventually lead to calcification, this will take much longer than in magnesium‐free medium.

**Fig. 5:**
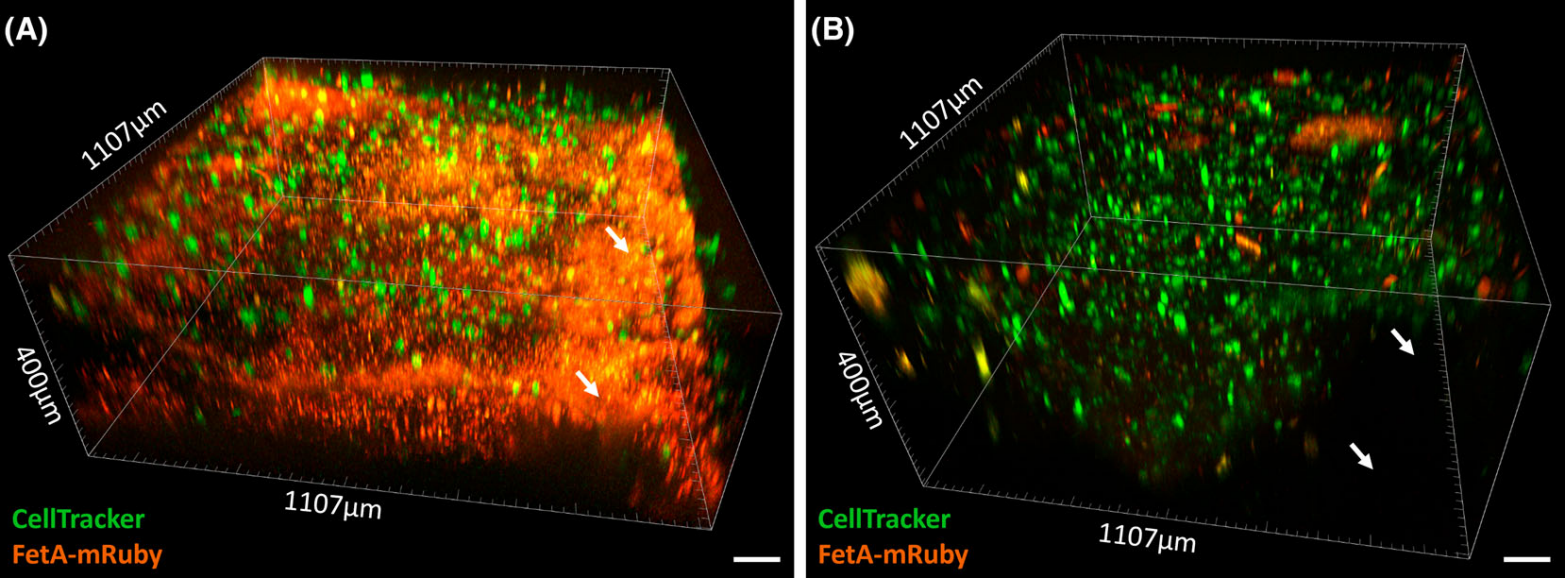
3D reconstruction of the samples imaged at the final day of the experiment (day 4), (A). without magnesium supplementation; (B). with added magnesium. Calcification medium without added magnesium caused significant calcification staining by Fetuin‐A‐mRuby (red), foremost on the textile scaffold (A, arrows), while additional magnesium severely suppressed red calcification staining, rendering the scaffold dark in microscopy images (B, arrows). Labeling: CellTracker Green CMFDA (green), Fetuin‐A‐mRuby (red). Scale bars = 100 μm.

The differences become even more evident in the 3D datasets (Fig. 5). In magnesium‐free medium, calcium deposits were abundant and distributed throughout the entire sample volume, with particularly pronounced calcification of the textile structure (Fig. 5A). In contrast, magnesium‐enriched samples showed only sparse and localized mineral accumulation, underscoring the strong inhibitory effect of magnesium on calcification (Fig. 5B).

The inhibitory effect of magnesium on calcification has been reported in previous studies [23], with proposed mechanisms suggesting that magnesium reduces spontaneous calcium phosphate precipitation [24], and also reduces or even stops cell‐mediated mineralization processes [25]. Our results are consistent with these findings and suggest that magnesium likely prevents calcium deposition on the textile reinforcement, which otherwise serves as an effective nucleation site under magnesium‐free conditions. We show that the flow chip setup in combination with multiphoton microscopy is capable to follow and distinguish the effect of magnesium on mineralization process, however further experiments would be required to statistically quantify the effects, using different concentration or shear stress conditions.

Most importantly, our results demonstrate a versatile platform that (a) applies physiologically relevant shear stress to cultured cells, (b) allows flexible adjustment of medium composition with the possibility of investigating boundary‐layer effects, and (c) enables longitudinal three‐dimensional imaging compatible with living samples at subcellular resolution. Its compatibility with nearly any type of biohybrid materials (e.g., vascular grafts, tissue‐engineered heart valves) or cell cultures and native tissues makes it unique in the field of chips used in biological research. All these features are achieved while maintaining sterility and cell viability during short (up to 30 min) transfers and imaging sessions outside the incubator. We believe this platform opens new avenues for studies requiring dynamic flow conditions, tunable media environments, and high‐resolution live‐cell imaging. While commercialization of this platform is currently not intended, the device in its current state could easily be given to researchers as a reusable, reliable way to perform such experiments in a standard cell culture laboratory.

### Limitations

It is important to note that the optical performance of this platform is inherently lower than that of conventional benchtop microscopy systems. First, the extended working distance of the objective is achieved at the expense of numerical aperture, limiting the theoretical resolution to approximately 0.6 μm laterally and 4 μm axially. Second, the complex excitation path introduces spherical and chromatic aberrations, which further degrade image quality.

One potential improvement would be to match the refractive index (RI) of the objective immersion medium to that of PMMA, thereby reducing refractive index mismatches at optical interfaces. In our setup, water immersion was used to facilitate cleaning of optical surfaces, with the resulting mismatch partially compensated by adjustment of the correction collar. While this approach improves signal quality in practice, it does not fully resolve the underlying optical inconsistencies.

To ensure the reliability of our observations, samples were re‐imaged after completion of the experiment taken out of the flow chip as a reference (‘ground truth’) essentially bypassing RI mismatch related aberrations, and the endpoint states were found to be in good agreement. We recommend a similar validation step when employing this workflow.

While our experiment clearly displays the SMCs' culture response to magnesium supplementation in calcifying conditions, the model has some important limitations from the perspective of vascular calcification.

First, although the experiments were conducted with a substantial volume of medium, no medium exchange was performed. As reported previously [13], medium exchange can be highly stressful, leading to significant cell loss. However, while avoiding medium exchange helps preserve sample viability, it may also result in gradual depletion of nutrients and staining components, eventually limiting further calcification detection. To mitigate this issue, the use of larger medium reservoirs or alternative strategies for gentle medium replenishment should be considered.

Second, in native vascular tissues, SMCs are not directly exposed to calcifying media as in our model but are separated from it by the endothelial cell (EC) layer. Therefore, SMC and EC cell co‐culture would give a better representation of the natural conditions.

Finally, the present study was performed using tissue‐engineered constructs, which are highly transparent and therefore well suited for deep optical imaging. Extending this approach to more optically dense samples, such as native vascular tissues, would likely reduce imaging depth. However, since calcification in such tissues often initiates and remains prominent at or near the surface, the proposed platform may still be well suited for investigating these processes.

## Conflict of interest

The authors declare no conflict of interest.

## Author contributions

VK wrote the manuscript and developed imaging protocols. ADM developed the flow chip design. VK and ADM designed the experiment and protocol. IC conducted trial experiments and optimized sample labeling. CB developed textile‐reinforced sample preparation workflow. SJ, WJ‐D, and MAMJZ supervised work done. All authors participated in the revision of the manuscript.

## Data Availability

The data that support the findings of this study are available from the corresponding author vytautas.kucikas@maastrichtuniversity.nl upon reasonable request.
